# Trends in HbA_1c_ thresholds for initiation of hypoglycemic agents: Impact of changed recommendations for older and frail patients

**DOI:** 10.1002/pds.5129

**Published:** 2020-09-21

**Authors:** Martina Ambrož, Sieta T. de Vries, Klaas Hoogenberg, Petra Denig

**Affiliations:** ^1^ Department of Clinical Pharmacy and Pharmacology University of Groningen, University Medical Center Groningen Groningen The Netherlands; ^2^ Department of Internal Medicine Martini Hospital Groningen The Netherlands

## Abstract

**Aims:**

Less strict glycated hemoglobin (HbA_1c_) thresholds have been recommended in older and/or frail type 2 diabetes (T2D) patients than in younger and less frail patients for initiating hypoglycemic agents since 2011. We aimed to assess trends in HbA_1c_ thresholds at initiation of a first hypoglycemic agent(s) in T2D patients and the influence of age and frailty on these trends.

**Materials and methods:**

The groningen initiative to analyze type 2 diabetes treatment (GIANTT) database was used, which includes primary care T2D patients from the north of the Netherlands. Patients initiating a first non‐insulin hypoglycemic agent(s) between 2008 and 2014 with an HbA_1c_ measurement within 120 days before initiation were included. The influence of calendar year, age, or frailty and the interaction between calendar year and age or frailty were assessed using multilevel regression analyses adjusted for confounders.

**Results:**

We included 4588 patients. The mean HbA_1c_ threshold at treatment initiation was 7.4% up to 2010, decreasing to 7.1% in 2011 and increasing to 7.4% in 2014. This quadratic change over the years was significant (*P* < 0.001). Patients aged 60 to 79 initiated treatments at lower HbA_1c_ and patients of different frailty at similar HbA_1c_ levels. The interaction between year and age or frailty was not significant (*P* > 0.05).

**Conclusions:**

HbA_1c_ thresholds at initiation of a first hypoglycemic agent(s) changed significantly over time, showing a decrease after 2010 and an increase after 2012. The HbA_1c_ threshold at initiation was not influenced by age or frailty, which is in contrast with recommendations for more personalized treatment.

**Key Points**

- The HbA_1c_ threshold at initiation of a first hypoglycemic agent(s) showed a temporary decrease to 7.1% in 2011 and increased to 7.4% in 2014.
- The trend in the HbA_1c_ threshold was not influenced by the patients' age or frailty, in contrast to recommendations for more personalized targets.
- Patients under 60 and over 80 years initiated treatment at significantly higher HbA_1c_ levels than patients aged 60 to 79 years.

## 1 INTRODUCTION

An important goal of type 2 diabetes (T2D) management is reducing the risk of complications by good control of blood glucose levels. This can be achieved with lifestyle changes but hypoglycemic agents have to be initiated when glucose control is insufficient. The success of T2D management is often monitored by regularly testing glycated hemoglobin (HbA_1c_) levels, which serve as a measure of chronic hyperglycemia.^1^ Several studies showed that the HbA_1c_ level at initiation of a first hypoglycemic agent is the main predictor of achieving early glycemic control.^2^, ^3^

Over the last decade, there have been several changes in treatment recommendations for patients with T2D (Supplementary Table 1 in Data S1). At first, achieving HbA_1c_ levels below 7% was recommended for most patients.^4^, ^5^, ^6^ Between 2008 and 2010, a performance measure assessing the percentage of patients achieving HbA_1c_ levels below 7% was introduced in primary care in the Netherlands.^7^, ^8^ Around 2009, several professional organizations started to advocate more personalized HbA_1c_ targets, particularly in elderly patients.^9^, ^10^ Diabetes guidelines started to recommend personalized HbA_1c_ treatment targets in 2011. This personalization was based on the patients' age and frailty. From 2011 onwards, guidelines recommended HbA_1c_ targets ≤7.0% for non‐frail patients younger than 70 years and between 7.0% and 8.5% for many patients older than 70 years with a longer diabetes duration and/or frail patients^9^, ^11^, ^12^, ^13^ (Supplementary Table 1 in Data S1). These targets are also considered as thresholds for initiating treatment. The extent to which these recommendations have led to more personalized initiation of hypoglycemic treatment in clinical practice is unknown.

The aim of our study was to investigate trends in HbA_1c_ thresholds at initiation of a first hypoglycemic agent(s) and the possible impact of more personalized treatment recommendations for older and frail patients with T2D. Given the introduction of performance measures and changes in treatment recommendations, we hypothesized that there would be a decrease in the overall mean HbA_1c_ thresholds in the period 2008 to 2014 but that first hypoglycemic agent(s) would be initiated at higher HbA_1c_ thresholds in older and frail patients after more personalized targets were introduced.

## 2 MATERIALS AND METHODS

### 2.1 Study design and population

This was a repeated cross‐sectional dynamic cohort study for the years 2008 to 2014. We used the data available from the GIANTT (www.giantt.nl) database, which contains anonymous primary care electronic medical records data from patients with T2D in the northern part of the Netherlands.

For each calendar year, patients were included if they had a confirmed diagnosis of T2D, were 18 years or older, and initiated treatment with a first hypoglycemic agent(s) in that year. This initiation was defined as a prescription for a non‐insulin hypoglycemic agent (anatomic therapeutic chemical [ATC] classification codes A10B) without a prescription for any hypoglycemic agent in the preceding 365 days. Included patients had to have at least 1 year of history in the GIANTT database before initiation of hypoglycemic treatment. We excluded patients without a documented HbA_1c_ level within 120 days before or on the day of treatment initiation. In addition, patients who had been diagnosed with T2D 10 or more years before treatment initiation and patients who initiated treatment with three or more hypoglycemic agents were excluded since it is unlikely that these patients were true initiators. An approval from the ethics committee is not needed for studies using anonymous medical records data in the Netherlands. We obtained an exemption letter from the University Medical Center Groningen Medical Ethics Review Board (reference number M19.235285).

### 2.2 Outcome variable

The primary outcome was the patient's most recent HbA_1c_ level in the 120 days before or on the day of a first hypoglycemic agent(s) initiation.

### 2.3 Explanatory variables

The following explanatory variables were included: calendar year of treatment initiation, age or frailty of the patient and the interaction between calendar year and age or frailty. Age was calculated on January 1 of the year in which the patient initiated treatment. We categorized age in four groups (<60 years, 60‐69 years, 70‐79 years, and ≥ 80 years old) based on the different cut‐offs observed among guidelines (Supplementary Table 1 in Data S1). Frailty was calculated using an electronic frailty index (eFI), which is based on International Classification of Primary Care (ICPC) coded diagnoses.^14^ We excluded diabetes from the eFI, thus focusing on differences in additional frailty. A higher number for the eFI indicates a higher degree of frailty. Since there are no validated clinical cut‐offs for the eFI, we categorized the scores in tertiles to compare low, medium, and high frailty patients.

### 2.4 Confounders

There are several patient characteristics available in the GIANTT database that can be associated with age or frailty and may affect the prescribers' decision to initiate a hypoglycemic agent. The following were included to correct for potential confounding: sex, duration of diabetes (0‐1 year, 2‐3 years, 4‐5 years, 6‐7 years, 8‐9 years), presence of dyslipidemia (defined as low density lipoproteins [LDL] ≥2.5 mmol/L), systolic blood pressure level (<140 mm Hg or ≥140 mm Hg), estimated glomerular filtration rate (eGFR; ≤60 mL/min or >60 mL/min), presence of albuminuria (albumin creatinine ratio ≥30 mg/g or albumin in 24 hours urine ≥300 mg), body mass index (BMI; <24.9 kg/m^2^, 25‐29.9 kg/m^2^ or ≥30 kg/m^2^), blood pressure lowering treatment (no treatment, 1 class, 2 classes, ≥3 classes), lipid lowering treatment (no treatment or ≥ 1 classes) and number of all other prescribed chronic medications at initiation (used as a continuous variable). The most recent laboratory values available in the year before or 7 days after initiation were used for these variables. BMI was calculated from weight and height based on the data in the last 5 years or in the year after initiation or extracted as provided BMI from the database when weight and/or height were not available. The eGFR was calculated from serum creatinine using the Modification of Diet in Renal Disease‐4 equation for the years 2008 and 2009, and using the Chronic Kidney Disease Epidemiology Collaboration equation from 2010 onwards, since the standard way of calculating eGFR in the Netherlands changed during the study period.^14^ In case serum creatinine was not available, the eGFR measurement was extracted as provided in the database. Prescribed chronic medication was assessed in the 120 days before or on the day of treatment initiation.

### 2.5 Missing data

No data for the explanatory variables were missing. When confounders had less than 20% of missing values, they were imputed using multiple imputation by chained equation (MICE).^15^ For albuminuria, more than 20% of patients had a missing value. These patients were assumed as not having albuminuria, since conducting this test in the study period was less common in patients without suspected kidney problems.

### 2.6 Analyses

Characteristics of included patients were analyzed descriptively per year. We conducted multilevel regression analyses with a two‐level random intercept model to account for patients being nested within general practices. First, using the empty model that includes only the outcome variable, we calculated the intraclass correlation coefficient (ICC). The ICC assesses the proportion of variance attributed to general practices. Second, we created the trend model by adding the calendar year and the confounders to the model to assess the overall trend over the years. We compared linear and non‐linear trend models using the Wald test to choose the best fitting final model. Next, we assessed the effect of age or frailty on these trends by adding the explanatory variables and the interaction between calendar year and age or frailty on HbA_1c_ levels at initiation in this trend model. To assess changes over time in separate age and frailty groups, additional multilevel analyses were conducted per subgroup. In these models, the Bonferroni method was used to correct for multiple testing, with a significance level of *P* < 0.0125 when testing for trends per age group and of *P* < 0.0167 when testing for trends per frailty group.

A sensitivity analysis was conducted in which the eFI was used as a continuous variable in the final model.

The analyses were conducted in Stata version 14 (Stata Corp., College Station, Texas).

## 3 RESULTS

**FIGURE 1:**
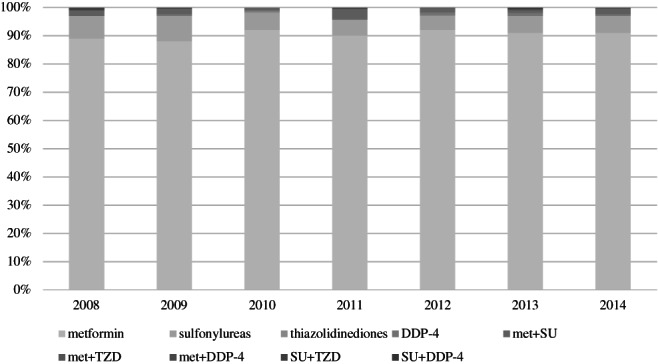
Type of first hypoglycemic agent(s) initiated from 2008 to 2014. DDP‐4, dipeptidyl peptidase‐4 inhibitor; met, metformin; SU, sulfonylurea; TZD, thiazolidinedione

We included 4588 patients who initiated a first hypoglycemic agent(s) between 2008 and 2014 (Table 1). The number of patients in each calendar year differed, whereas the patient characteristics were similar over the years (Supplementary Table 2 in Data S1). Around 90% of patients initiated treatment with metformin (Figure 1). The use of sulfonylureas slightly decreased over the years from 8% to 6%, mostly on the account of the newer medication that became available in this time period. Complete data were available for 74% of the patients.

**TABLE 1:** Characteristics of patients included in the analysis (N = 4588)

**Number of patients in source population; N**	
2008 (N = 15 086)	345
2009 (N = 18 130)	536
2010 (N = 20 995)	732
2011 (N = 24 059)	744
2012 (N = 26 319)	781
2013 (N = 27 342)	670
2014 (N = 30 450)	780
**Females; N (%)**	2289 (50)
**Age in years; N (%)**	
<60	1561 (34)
60‐69	1478 (32)
70‐79	1086 (24)
≥80	463 (10)
**Frailty in electronic Frailty Index score; N (%)**	
0‐0.03	1679 (37)
0.06‐0.08	1551 (34)
0.11‐0.30	1358 (30)
**Glycated hemoglobin A**_**1c**_ **at initiation in %; mean ± SD**	7.3 **±** 1.1
**Fasting glucose; mean ± SD** ^a^	8.6 ± 2.2
**Diabetes duration; N (%)**	
0‐1 years	1522 (33)
2‐3 years	1384 (30)
4‐5 years	881 (19)
6‐7 years	523 (11)
8‐9 years	289 (6)
**Systolic blood pressure ≥ 140 mm Hg; N (%)** ^b^	2263 (54)
**BMI in kg/m** ^**2**^ **; N (%)** ^c^	
<24.9	521 (12)
25‐29.9	1658 (39)
≥30	2101 (49)
**Dyslipidemia; N (%)** ^d^	2631 (65)
**eGFR ≤ 60 mL/min/1.73 m2; N (%)** ^e^	680 (16)
**Albuminuria (%)** ^f^	52 (1)
**Number of chronic medication at initiation; mean ± SD**	4.1 ± 2.9
**Blood pressure lowering treatment at initiation; N (%)**	
No treatment	1477 (32)
1 class	1124 (25)
2 classes	1077 (23)
3 or more classes	910 (20)
**Treated with a lipid lowering drug; N (%)**	2679 (58)

^a^ Fasting glucose: 1 170 (25.5%) missing values.

^b^ Systolic blood pressure: 399 (8.7%) missing values.

^c^ Body mass index (BMI): 308 (6.7%) missing values.

^d^ Low‐density lipoprotein (LDL) cholesterol: 568 (12.4%) missing values.

^e^ Estimated glomerular filtration rate (eGFR): 430 (9.4%) missing values.

^f^ Albuminuria: 2353 (51.3%) missing values.

### 3.1 Trends in HbA_1c_ thresholds

**FIGURE 2:**
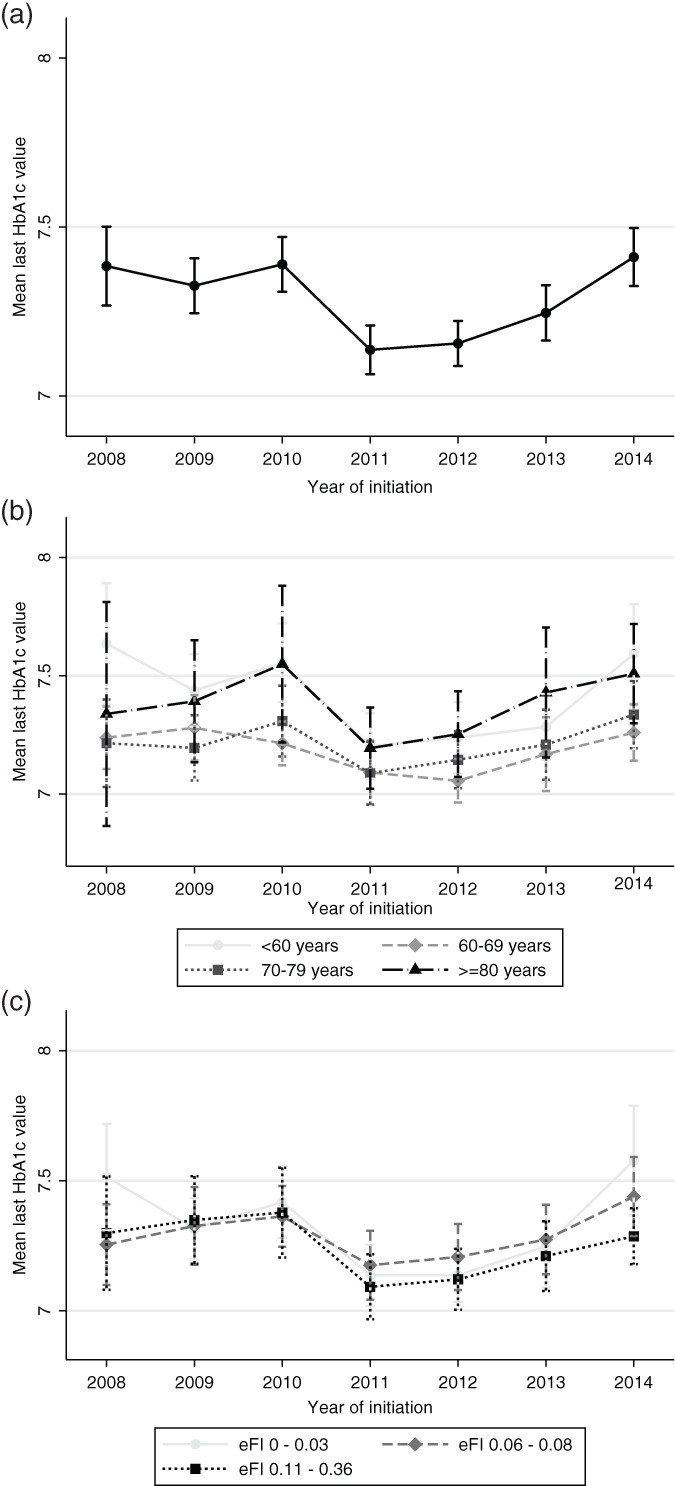
Mean last glycated haemoglobin A1c (HbA_1c_) levels with 95% confidence intervals before/at initiation of the first hypoglycemic agent(s) from 2008 to 2014 in (a) the whole population, (b) different age groups and (c) different frailty groups. eFI: electronic frailty index

The mean HbA_1c_ level before or at initiation of a first hypoglycemic agent(s) changed quadratically over the years (β(year) = −0.236, 95% CI –0.334, −0.138, *P* < 0.001; β(year^2^) = 0.021, 95% CI 0.012, 0.030, *P* < 0.001; joint p using Wald test <0.001; Figure 2A). A stable HbA_1c_ level at treatment initiation of around 7.4% was observed between 2008 and 2010. This was followed by a decrease to 7.1% in 2011 and a rise thereafter to 7.4% in 2014 (Figure 2A, Supplementary Table 2 in Data S1).

Of the total variation in HbA_1c_ level at treatment initiation, 6.4% was explained by differences between general practices (ICC = 0.064).

### 3.2 Age and frailty

Patients between 60 and 79 years initiated treatment at significantly lower HbA_1c_ levels than younger or older patients (Table 2). The drop in HbA_1c_ thresholds between 2010 and 2011 was visible in all age groups, as was the rise after 2012 (Figure 2B). Although some differences in trends between the age groups can be observed after 2012, the interaction between age and calendar year was not statistically significant (Table 2). In the analysis per age group, the HbA_1c_ threshold changed significantly over the years in patients younger than 60 years old (β(year) = −0.407, 95% CI –0.608, −0.205, *P* < 0.001; β(year^2^) = 0.036, 95% CI 0.017, 0.055, *P* < 0.001; joint p using Wald test <0.001) and aged 60 to 69 years (β(year) = −0.216, 95% CI –0.360, −0.072, *P* = 0.003; β(year^2^) = 0.019, 95% CI 0.005, 0.033, *P* = 0.007; joint *P* using Wald test =0.008), whereas this trend was not significant in other two groups.

**TABLE 2:** Influence of calendar year and age or frailty on glycated hemoglobin A_1c_ (HbA_1c_) thresholds (multilevel analysis)

	β	95% CI	*P*	
**AGE** ^a^				
Calendar year	−0.241	−0.338, −0.143	<0.001	<0.001^b^
Calendar year^2^	0.021	0.012, 0.031	<0.001	
Age <60 years	−0.063	−0.187, 0.061	0.320	
Age 60–69 years	−0.256	−0.374, −0.138	0.000	
Age 70‐79 years	−0.185	−0.301, −0.069	0.002	
Age ≥80 years	Reference group			
Interaction year*age	None are significant			
**FRAILTY** ^c^				
Calendar year	−0.223	−0.321, −0.125	<0.001	<0.001^b^
Calendar year^2^	0.020	0.011, 0.030	<0.001	
Frailty 0‐0.03	−0.005	−0.090, 0.081	0.917	
Frailty 0.06‐0.08	0.057	−0.021, 0.134	0.151	
Frailty 0.11‐0.36	Reference group			
Interaction year*frailty	None are significant			

*Note*: The intraclass correlation coefficient (ICC) calculated from the empty model was 0.064.

^a^ The age model was adjusted for sex, duration of diabetes, number of chronic medication at initiation, number of antihypertensive drug classes, systolic blood pressure, lipid lowering therapy, presence of albuminuria, presence of dyslipidemia, estimated glomerular filtration rate, and BMI.

^b^ Joint significance of calendar year and calendar year^2^ using Wald test.

^c^ The frailty model was adjusted for sex, systolic blood pressure, duration of diabetes, number of antihypertensive drug classes, and lipid lowering therapy.

All frailty groups initiated hypoglycemic treatment at similar HbA_1c_ thresholds (Figure 2C; Table 2). The interaction between frailty and calendar year was not significant. In the analysis per frailty group, the HbA_1c_ threshold changed significantly over the years in the least frail group (β(year) = −0.345, 95% CI –0.515, −0.176, *P* < 0.001; β(year^2^) = 0.032, 95% CI 0.016, 0.049, *P* < 0.001; joint *P* using Wald test <0.001), but this trend was not significant in the other two groups. The sensitivity analysis, using frailty index as a continuous variable, showed similar non‐significant results (Supplementary Table 3 in Data S1).

## 4 DISCUSSION

The mean HbA_1c_ level at initiation of a first hypoglycemic agent(s) decreased after 2010 and increased after 2012 until the end of our study period in 2014. Surprisingly, there were no differences in the trends for patients of different ages or frailty between 2008 and 2014.

The rising trend in HbA_1c_ level at treatment initiation after 2012 is not in line with our hypothesis, since we expected a decrease in the overall HbA_1c_ threshold throughout the study period. It is, however, in line with a recent study conducted in Denmark, which assessed the trends in pre‐treatment HbA_1c_ levels between 2000 and 2017, where a similar decreasing pattern up to 2011 with a slight increase thereafter was observed.^16^ Other studies have looked at trends in proportions of patients achieving target levels, showing either increases or non‐significant changes over time.^17^, ^18^, ^19^ An intriguing finding of our study was that a drop in HbA_1c_ levels was particularly seen between 2010 and 2011. This may be due to policy changes in the Netherlands. In 2008, performance measures were introduced as informative indicators for benchmarking the general practitioners (GPs) on achieving low targets in diabetes patients. In our study region, additional education and support was offered around 2010 to the GPs to improve their performance. We did not expect, however, that the HbA_1c_ would increase after 2012. This could indicate that the performance measures and other activities only had a temporary effect.

Our study showed no differences in HbA_1c_ levels at hypoglycemic treatment initiation in patients of different ages. This is not in line with our hypothesis and recommendations of using higher HbA_1c_ targets for older T2D patients after 2011 (Supplementary Table 1). Surprisingly, the youngest and the oldest patients initiated treatment at similar slightly higher HbA_1c_ levels. On the one hand, this could be due to more delay in diagnosing diabetes in younger as compared to older patients, who are more actively monitored. This would lead to higher HbA_1c_ levels at diagnosis and subsequently at treatment initiation. It has indeed been shown that the HbA_1c_ levels at diagnosis were higher in younger than in older patients.^20^, ^21^ On the other hand, it was found that the time to initiation of a hypoglycemic agent increased with advancing age.^20^, ^21^ Thus, the HbA_1c_ level at treatment initiation can be higher in younger patients because of a delay in diagnosis, while it can be higher in older patients because of a delay in treatment initiation. Interestingly, the HbA_1c_ level at initiation increased after 2012 in all age groups, with this increase being the highest in patients younger than 60 years. We can only speculate about the possible explanations. It could be that either the GPs or the patients prefer to try lifestyle changes for a longer period at a younger age, leading to higher HbA_1c_ levels when deciding to initiate medication. It could also be that GPs became less strict in all patients because potential overtreatment for diabetes has been gaining a lot of attention in the last decade.^7^

Similar to age, there were no significant differences between patients with different levels of frailty. Frailty has not been used in previous analyses of hypoglycemic treatment patterns, however, a recent study observed that patients with three or more comorbidities were more likely to have a tighter glycemic control than patients with no or only one comorbidity.^22^ We conducted a post‐hoc analysis using the number of chronic medications at initiation as a proxy for frailty and found that patients receiving less than four (median) chronic medications initiated treatment at significantly higher HbA_1c_ levels when compared to four or more chronic medications (Supplementary Figure 1 and Supplementary Table 4 in Data S1). Furthermore, the observed increase after 2012 particularly in patients prescribed less medication is again unexpected. These results do not support our hypothesis that less strict treatment thresholds were applied for frail patients. A possible explanation could be that frailty measured with the eFI score—or with the number of chronic medication—is not fully applicable or fitting in clinical practice. The eFI was comparable to the Groningen Frailty Index in the previous studies^14^ but it might not be in line with the GPs' perception of the patient's status. Also, frailty can easily be overlooked in practice due to its subtle manifestations and a lack of consensus on how best to assess it.^23^ In addition, specific factors such as life expectancy, functional dependency, and risk of hypoglycemia, which are mentioned in relation to personalized treatment targets, may contribute more to the prescribers' decisions to initiate treatment than frailty in general.

Our study provides important insights in prescribing trends and suggests that trends in initiation of a first hypoglycemic agent(s) may not be fully in accordance to changes in recommendations towards more personalized treatment. The lack of differentiation between patients of different ages and frailty is of concern. The increase in HbA_1c_ thresholds after 2012 in older patients who do not benefit from tight control is encouraging but this trend was not observed in the most frail patients. Moreover, this trend appeared stronger in the youngest age group, where it is unfavorable and indicates undertreatment of younger and fit patients for whom the disease is not well controlled and can lead to preventable complications. Possible explanations for this observation should be studied further.

Implementing personalized treatment in diabetes may require further support. A study conducted in the period 2010 to 2012 in seven European countries, in which physicians were first trained to set personalized targets, showed that the targets they set for older patients only marginally deviated from the traditional HbA_1c_ target of 7%. Neither age, duration of diabetes, presence of polypharmacy or frailty had a significant impact on the targets set.^24^ These results suggest that only issuing new guidelines or providing a training might not be enough to implement personalized diabetes treatment in practice. It has been proposed to offer additional tools or algorithms to support clinical decision‐making, which may help in setting more personalized targets in practice.^25^, ^26^, ^27^

The strength of our study is inclusion of a large number of patients using real‐world data from primary care. It is also a first study to examine trends in HbA_1c_ level at initiation of a first hypoglycemic agent(s) and to compare patients of different ages and frailty. Our study has some limitations. First, the number of GPs included in each calendar year fluctuates. Since only little variation was explained at practice level, we do not expect that this affected our conclusions. Second, approximately 10% of patients initiating hypoglycemic therapy were excluded from our analysis because they initiated treatment with insulin. Although it is unlikely that these patients were true initiators, other studies have shown similar rates of initial therapy with insulin in patients with T2D.^28^, ^29^ Therefore, we conducted a post‐hoc analysis including patients who initiated treatment with insulin, which revealed similar results (data not shown). Third, the observed time between diabetes diagnosis and treatment initiation was quite long for some patients. This could be due to persisting with lifestyle changes for several years before initiating medication treatment. We have to acknowledge, however, that some GPs may have included patients with early stages of diabetes or prediabetes in our cohort. We therefore conducted another post‐hoc analysis including only patients with diabetes duration of 5 years or less (N = 3412), showing similar results (data not shown). Finally, we had some missing data but these were imputed using multiple imputation to reduce possible bias. Frailty, however, was probably underestimated due to incomplete coding of ICPC diagnoses in electronic medical records.

In conclusion, the observed HbA_1c_ thresholds at initiation of a first hypoglycemic agent(s) changed significantly over time, showing a decrease after 2010 followed by an increase after 2012. This quadratic trend was not influenced by patients' age or frailty, which is in contrast with changed recommendations for more personalized treatment targets in the study period. More research is needed to determine factors influencing decisions to initiate or refrain from initiating hypoglycemic treatment in general practice, particularly for frail patients. Furthermore, the reasons for initiating diabetes treatment at increasingly higher HbA_1c_ levels in relatively young patients should be further investigated.

## CONFLICT OF INTEREST

The authors have no conflicts of interest to declare.

## AUTHOR CONTRIBUTIONS

M.A. contributed to the development and formulation of the research question, conducted the analysis, contributed to the interpretation of data, wrote the manuscript, and reviewed and edited the manuscript. S.T.d.V. contributed to the development and formulation of the research question, conducted the analysis, contributed to the interpretation of data, and reviewed and edited the manuscript. K.H. contributed to the development and formulation of the research question, the interpretation of data, and reviewed and edited the manuscript. P.D. contributed to the development and formulation of the research question, development of the analysis, the interpretation of data, and reviewed and edited the manuscript.

## ETHICS STATEMENT

The authors state that no ethical approval was needed.

## Supporting information


**Data S1**. Supporting Information.
