# Supplementary material for: Trends in HbA1c thresholds for initiation of hypoglycemic agents: Impact of changed recommendations for older and frail patients
Source: Pharmacoepidemiol Drug Saf. 2020 Sep 21;30(1):37–44. doi: 10.1002/pds.5129 (PMC7756585; doi:10.1002/pds.5129)
Supplement: Supplementary file 1 — Data S1. Supporting Information. [file PDS-30-37-s001.docx]

**Supplementary appendix**

**Supplementary table 1**: Overview of changes in Dutch, European and Global type 2 diabetes (T2D) protocol and guideline recommendations regarding glycated haemoglobin A1c (HbA1c) target levels

|  | **Year** | | **HbA1c**  **target** | | **Conditions** | | |
| --- | --- | --- | --- | --- | --- | --- | --- |
| **NATIONAL** | |  | | | |  |  |
| Diabeteszorg hoogbejaarden^1^ | 2009 | | <7.5%  <8%  >8% | >70 years  >80 years  if lifetime expectancy <5 years | | | |
| NHG T2D ^2,3^ | 2006 | | <7% | all patients | | | |
| Verenso ^4^ | 2013 | | ≤7%  ≤7%  ≤7.5%  ≤8% | <70 years  >70 years, treated with only lifestyle or metformin  >70 years, treated with more than metformin and diabetes duration <10 years  >70 years, treated with more than metformin and diabetes duration >10 years | | | |
|  | 2011 | | <8.5% | frail elderly (high to very high age, chronically ill, restrictions, multiple morbidity), life expectancy <6 years | | | |
| **EUROPEAN** | | | | | | | |
| ESC/EADS ^5^ | 2007 | | <6.5% | all patients | | | |
| EU working party for older T2D patients ^6^ | 2011 | | 7 – 7.5%  7.6 – 8.5% | >70 years without major comorbidities  >70 years and frail (dependent, multisystem disease, care home residents) | | | |
| **GLOBAL** |  |  |  |  |  |  |  |
| Global T2D guideline ^7,8^ | 2006  2012 | | <6.5%  <7%  7 – 7.5% | any improvement is beneficial; higher targets are acceptable if there is a high risk of hypoglycemia (insulin, sulfonylureas)  higher target is acceptable in presence of hypoglycemia, comorbidities or limited life expectancy  >70 years | | | |
| Managing older T2D patients ^9^ | 2013 | | 7 – 7.5%  7.5 – 8%  <8.5% | >60 years and  functionally independent  functionally dependent  functionally dependent and frail | | | |

^1^ Verhoeven S, Bilo H, van Hateren K, Houweling ST, Kleefstra N, van Meeteren J. Protocol: Diabeteszorg hoogbejaarden in verzorgings- en verpleeghuizen [Protocol: Diabetes care for the very elderly in care and nursing homes]. Diabetes Specialist 2009.

^2^ Rutten G, De Grauw W, Nijpels G, Goudswaard AN, Uitewaal P, Van der Does F, et al. NHG-Standaard Diabetes mellitus type 2 (tweede herziening) [NHG standard diabetes mellitus type 2 (second review)]. Huisarts en wetenschap 2006;49(3):137-152.

^3^ Rutten G, De Grauw W, Nijpels G, Houweling ST, Van de Laar, F A, Bilo HJ, et al. NHG-Standaard Diabetes mellitus type 2 (derde herziening) [NHG standard diabetes mellitus type 2 (third review)]. Huisarts Wet 2013;10(56):512-525.

^4^ Verenso Richtlijnwerkgroep. Multidisciplinaire Richtlijn Diabetes. Verantwoorde Diabeteszorg bij Kwetsbare Ouderen Thuis en in Verzorgings of Verpleeghuizen. Deel 1. [Multidisciplinary Guideline Diabetes. Responsible Diabetes Care in Vulnerable Elderly at Home and in Residential Care or Nursing Homes. Part 1]. 2011:1-140.

^5^ Rydén L, Standl E, Bartnik M, Van den Berghe G, Betteridge J, de Boer M, et al. Guidelines on diabetes, pre-diabetes, and cardiovascular diseases: executive summary: The task force on diabetes and cardiovascular diseases of the European Society of Cardiology (ESC) and of the European Association for the Study of Diabetes (EASD). European heart journal 2007;28(1):88-136.

^6^ Sinclair, AJ, Paolisso G, Castro M, Bourdel-Marchasson I, Gadsby R, Rodriguez Mañas L. European diabetes working party for older people 2011 clinical guidelines for type 2 diabetes mellitus. Executive summary. Diabetes & Metabolism 2011;37:S27-S38.

^7^ IDF Clinical Guidelines Task Force. Global guideline for type 2 diabetes: recommendations for standard, comprehensive, and minimal care. Diabetic Medicine 2006;23(6):579-593.

^8^ IDF Clinical Guidelines Task Force. Global guideline for type 2 diabetes. Brussels, Belgium: International Diabetes Federation; 2012.

^9^ IDF Working Group. Managing older People with type 2 diabetes. Brussels, Belgium: International Diabetes Federation; 2013.

|  | **Supplementary table 2:** Characteristics of included patients over the years | | | | | | | | |
| --- | --- | --- | --- | --- | --- | --- | --- | --- | --- |
|  | | **2008** | **2009** | **2010** | **2011** | **2012** | **2013** | **2014** |  |
| **Number of patients** | | 345 | 536 | 732 | 744 | 781 | 670 | 780 |  |
| **Females; N (%)** | | 165 (48) | 257 (48) | 394 (54) | 360 (48) | 401 (51) | 319 (48) | 393 (50) |  |
| **Age in years; N (%)** | |  |  |  |  |  |  |  |  |
| < 60 | | 125 (36) | 195 (36) | 261 (36) | 235 (32) | 249 (32) | 263 (39) | 233 (30) |  |
| 60 – 69 | | 118 (34) | 187 (35) | 229 (31) | 233 (31) | 268 (34) | 193 (29) | 250 (32) |  |
| 70 - 79 | | 76 (22) | 117 (22) | 172 (24) | 195 (26) | 179 (23) | 155 (23) | 192 (25) |  |
| ≥ 80 | | 26 (8) | 37 (7) | 70 (10) | 81 (11) | 85 (11) | 59 (9) | 105 (13) |  |
| **Frailty in electronic Frailty Index score; N (%)** | | |  |  |  | | |  |  |
| 0 – 0.03 | | 160 (46) | 238 (44) | 301 (41) | 281 (38) | 281 (36) | 221 (33) | 197 (25) |  |
| 0.06 – 0.08 | | 112 (32) | 186 (35) | 242 (33) | 247 (33) | 263 (34) | 241 (36) | 260 (33) |  |
| 0.11 – 0.36 | | 73 (21) | 112 (21) | 189 (26) | 216 (29) | 237 (30) | 208 (31) | 323 (41) |  |
| **HbA1c at initiation in %; mean ± SD** | | 7.4 ± 1.1 | 7.3 ± 1.0 | 7.4 ± 1.1 | 7.1 ± 1.0 | 7.2 ± 0.9 | 7.2 ± 1.1 | 7.4 ± 1.2 |  |
| **Fasting glucose; mean ± SD** | | 8.5 ± 2.0 | 8.5 ± 2.1 | 8.4 ± 2.2 | 8.4 ± 2.1 | 8.4 ± 2.1 | 8.8 ± 2.4 | 9.0 ± 2.7 |  |
| **Diabetes duration; N (%)** | |  |  |  |  |  |  |  |  |
| 0 – 1 years | | 125 (36) | 178 (33) | 287 (39) | 282 (38) | 257 (33) | 179 (27) | 203 (26) |  |
| 2 – 3 years | | 119 (34) | 183 (34) | 196 (27) | 186 (25) | 257 (33) | 215 (32) | 228 (29) |  |
| 4 – 5 years | | 54 (16) | 93 (17) | 146 (20) | 148 (20) | 131 (17) | 123 (18) | 186 (24) |  |
| 6 – 7 years | | 28 (8) | 54 (10) | 63 (9) | 94 (13) | 95 (12) | 94 (14) | 95 (12) |  |
| 8 – 9 years | | 19 (6) | 28 (5) | 40 (5) | 34 (5) | 41 (5) | 59 (9) | 68 (9) |  |
| **3Systolic blood pressure ≥140 mmHg; N (%)** | | 170 (59) | 281 (60) | 331 (53) | 383 (56) | 398 (53) | 315 (49) | 385 (52) |  |
| **BMI in kg/m^2^; N (%)** | |  |  |  |  |  |  |  |  |
| < 24.9 | | 36 (13) | 61 (13) | 86 (13) | 79 (11) | 101 (13) | 77 (12) | 81 (11) |  |
| 25 – 29.9 | | 104 (37) | 174 (38) | 254 (37) | 274 (39) | 309 (41) | 255 (39) | 288 (38) |  |
| > 30 | | 138 (50) | 221 (49) | 338 (50) | 353 (50) | 350 (46) | 321 (49) | 380 (51) |  |
| **Dyslipidaemia; N(%)** | | 162 (59) | 270 (61) | 415 (68) | 452 (69) | 460 (64) | 413 (67) | 459 (65) |  |
| **eGFR ≤ 60** **mL/min/1.73m^2^; N (%)** | | 61 (21) | 129 (27) | 88 (14) | 97 (14) | 101 (14) | 94 (15) | 110 (15) |  |
| **Albuminuria; N (%)** | | 3 (1) | 6 (1) | 5 (1) | 10 (1) | 15 (2) | 3 (0) | 10 (1) |  |
| **N of chronic medication at initiation; mean ± SD** | | 3.8 ± 2.7 | 4.2 ± 3.0 | 4.1 ± 2.8 | 4.1 ± 3.0 | 4.1 ± 3.0 | 4.0 ± 2.8 | 4.2 ± 3.1 |  |
| **Blood pressure lowering treatment at initiation; N (%)** | | | | | |  | |  |  |
| No treatment | | 115 (33) | 166 (31) | 229 (31) | 228 (31) | 255 (33) | 225 (34) | 259 (33) |  |
| 1 class | | 87 (25) | 115 (21) | 193 (26) | 196 (26) | 184 (24) | 163 (24) | 186 (24) |  |
| 2 classes | | 78 (23) | 150 (28) | 148 (20) | 182 (24) | 181 (23) | 154 (23) | 184 (24) |  |
| 3 or more classes | | 65 (19) | 105 (20) | 162 (22) | 138 (19) | 161 (21) | 128 (19) | 151 (19) |  |
| **Treated with a lipid lowering drug; N (%)** | | 205 (59) | 305 (57) | 438 (60) | 415 (56) | 474 (61) | 399 (60) | 443 (57) |  |

HbA1c: glycated haemoglobin; BMI: body mass index; eGFR: estimated glomerular filtration rate

**Supplementary table 3**: Influence of calendar year and frailty on glycated haemoglobin A1c (HbA1c) thresholds (multilevel analysis), using frailty index as a continuous variable

|  | **β** | **95% CI** | **P** | |
| --- | --- | --- | --- | --- |
| **FRAILTY** |  |  |  | |
| Calendar year | -0.223 | -0.321, -0.125 | <0.001 | <0.001^$^ |
| Calendar year^2^ | 0.020 | 0.011, 0.029 | <0.001 |  |
| Frailty | 0.079 | -0.487, 0.644 | 0.785 | |
| Interaction year*frailty | not significant | | | |
| The model was adjusted for sex, duration of diabetes, number of antihypertensive drug classes, lipid lowering therapy and systolic blood pressure | | | | |

^$^joint significance of calendar year and calendar year^2^ using Wald test

**Supplementary figure 1:** Mean last glycated haemoglobin A1c (HbA1c) level with 95% confidence intervals (CIs) before or at initiation of a first hypoglycaemic agent(s) through the years in patients with different number of chronic medication at initiation.

**Supplementary table 4**: Multilevel analysis of number of chronic medication at initiation

|  | **β** | **95% CI** | **P** | |
| --- | --- | --- | --- | --- |
| Calendar year | -0.242 | -0.341, -0.144 | <0.001 | <0.001^$^ |
| Calendar year^2^ | 0.022 | 0.012, 0.031 | <0.001 |  |
| 4 or less chronic medication | reference group | | | |
| More than 4 chronic medication | -0.105 | -0.167, -0.042 | 0.001 | |
| Interaction calendar year*N of medication | not significant | | | |

The model was adjusted for sex, duration of diabetes, presence of albuminuria, presence of dyslipidaemia, systolic blood pressure, estimated glomerular filtration rate and body mass index.

^$^joint significance of calendar year and calendar year^2^ using Wald test
